# Supplementary material for: Pre-Procedural Lumbar Neuraxial Ultrasound—A Systematic Review of Randomized Controlled Trials and Meta-Analysis
Source: Healthcare (Basel). 2021 Apr 17;9(4):479. doi: 10.3390/healthcare9040479 (PMC8072649; doi:10.3390/healthcare9040479)
Supplement: Supplementary file 1 [file healthcare-09-00479-s001.zip › healthcare-1161603-supplementary.pdf]

SUPPLEMENTARY TABLES AND FIGURES

Supplementary Table 1: Search Strategy

The research was performed using Medline, Science Direct, Scopus, and Cochrane Central databases**.** The following terms were included:

“Ultrasound” OR “ultrasonography”
AND
"epidural" OR "peridural" OR "subarachnoid" OR "spinal" OR “lumbar” OR “neuraxial”
AND
"analgesia" OR “anesthesia” OR “space” OR “puncture”

The research was limited by language (English only), Publication date (from 01/01/1980 to 31/12/2018), age of participants (adults) and availability of full text article. Congress abstracts, pain therapy articles, thoracic epidural articles were excluded. The database’s filters were used in order to follow the research strategy.

Pubmed (date: 01/07/2019)
(((Ultrasound[Title/Abstract] OR ultrasonography[Title/Abstract])) AND (epidural[Title/Abstract] OR peridural[Title/Abstract] OR spinal[Title/Abstract] OR subarachnoid[Title/Abstract] OR lumbar[Title/Abstract] OR neuraxial[Title/Abstract])) AND (anesthesia or analgesia or puncture or space) PublicationDate Filters: Humans; Adult: 19+ years

969 results

Science Direct (date: 01/07/2019)
“Ultrasound” OR “ultrasonography”) AND ("epidural" OR "peridural" OR "subarachnoid" OR "spinal") AND ("analgesia" OR “anesthesia” OR “space”)

245 results (research articles only)

Cochrane CENTRAL (date: 01/07/2019)
(Ultrasound OR ultrasonography):ti,ab,kw AND (epidural or peridural or lumbar or spinal or subarachnoid or neruraxial):ti,ab,kw AND (space or anesthesia or analgesia or puncture):ti,ab,kw (Word variations have been searched)

794 results

Scopus (date: 01/07/2019)
(TITLE-ABS-KEY(ultrasound or ultrasonography) AND TITLE-ABS-KEY(epidural or peridural or spinal or subarachnoid or lumbar or neuraxial)AND TITLE-ABS-KEY(anesthesia or analgesia or space or puncture)) AND DOCTYPE(ar) AND PUBYEAR > 1979 AND (LIMIT-TO (LANGUAGE,"English")) AND (LIMIT-TO (SRCTYPE,"j")) AND (LIMIT-TO (SUBJAREA,"MEDI") OR LIMIT-TO (SUBJAREA,"HEAL"))

2137 results

Supplementary Table 2: Additional characteristics of included trials

Author, Year; Country	Patient Population (n)	Technique (Methods)	Main Findings
**Spinal Anesthesia**			
**Abdelhamid, 2013; Egypt**	Adult unspecified (n=90, 45 each)	Spinal at L4-5 (US vs. LM group)	First attempt success 80% in US group and 37.8% in LM group (p<0.001). Needle redirection attempts were 15.6% in US group and 35.5% in LM group (p=0.002). Procedure time was longer in the US group (8.7 ±1.0 *vs.* 5.4 ± 0.4, p<0.001). Patient's satisfaction was higher in US group (95.6%) than the LM group (77.8%)(p=0.038).
**Ansari, 2014; UAE**	Obstetrics CS (n=150, 75 each)	Spinal at L3-4 or L4-5 (US vs. LM group)	Procedure time, number of insertions/redirections and first attempt success was equal among groups. No differences in complication rates among groups.
**Chin, 2011; Canada**	Orthopedic difficult spine (n=120, 60 each) 1. poorly palpable or impalpable spinous processes and BMI>35<br>2. moderate to severe lumbar scoliosis<br>3. previous lumbar spinal surgery involving removal of 2 or more spinous processes of L2- to L5	Spinal (US vs. LM group)	First-attempt success rate higher in US group (65% *vs.* 32%; *P* < 0.001). Number of insertions (US: 1 [1–2] *vs.* LM: 2 [1– 4]; p< 0.001) and number of redirections (US: 6 [1–10] *vs.* LM: 13 [5–21];p<0.003).) were higher in the LM group. Total procedure time was longer in the US group but needling time was shorter (US: 5.0±4.9 *vs.* LM: 7.3±7.6 min; p<0.038).

**Creaney, 2016; Ireland**	Obstetrics, CS, difficult spine (impalpable spinous processes) (n=20, 10 each)	Spinal L3-4 (US vs. LM group)	Fewer needle redirections in the US group (median 3 [IQR 1.8–3.2]) compared to the LM group (median 5.5 [IQR 3.2–7.2] (P=0.03)). More time was required to locate the needle insertion point in the US group (US: 91.8 ± 30.8 s *vs*. LM: 32.6 ± 11.4 s, P <0.001). There was no difference in the total procedural time between groups (US 191.8 ± 49.4 s *vs*. LM 192 ± 110.9 s, P=0.99).
**Dhanger, 2017; India**	Obstetrics CS (n=100, 50 each)	Spinal L3-4 (US vs. LM group)	Number of insertions (1.04 ± 0.19 *vs*. 1.97 ± 0.77), number of redirections in the same intervertebral space (1.26 ± 0.44 *vs*. 1.90 ± 0.51) and total procedure time (31.90 ± 6.30 *vs*. 51.80 ± 12.28 s) were significantly less in US group as compared to the LM group. Identification time was significantly longer in the US group (56.70 ± 13.08 s) compared to the LM group (47.10 ± 10.45 s).
**Ekinci, 2017; Turkey**	Obstetrics, difficult spine (n=64, 32 each) grade 2: Spinous processes cannot be palpated, interspinous spaces are not evident, and vertebral column can be palpated on the midline or outside the midline, and 3: Spinous processes cannot be palpated, interspinous spaces are not evident, and vertebral column cannot be palpated.	Spinal (US vs. LM group)	Number of needle insertions in the US group was significantly lower than the LM group (1.19±0.47 *vs*. 1.84 ± 0.85; p<0.001. No difference between the groups regarding number of redirections, levels attempted, and procedure time. First attempt success rate on a single skin puncture in US (84.4%) group was higher than the LM group ((84.4% *vs*. 40,6%; p=.001). No difference between the groups regarding first direction success.
**Li, 2018; China**	Obstetrics elective CS, obese (BMI > 30 kg/m2) (n=80, 40 each)	Spinal (US vs. LM group)	Higher first-attempt success rate for the US group (87.5% *vs*. 52.5%; p=0.001), fewer cases requiring >10 needle redirections (1 *vs*. 17; p<0.001), and fewer needle insertions and redirections (p<0.001). No difference in time taken to identify the needle insertion site. Needling time and total procedure time were significantly longer in the LM group (p<0.001). Patient satisfaction scores were significantly higher in the US group (p=0.001). For BMI 30-35 kg/m^2, no difference in first-attempt success rate,

			number of cases with >10 needle passes, spinal injection time or total procedure time. For BMI 35-43 kg/m^2, the US group had a significantly higher first-attempt success rate, p≤0.041), fewer cases with >10 needle passes (p≤0.01), and shorter procedure times, including the time required to identify the needle insertion site (p<0.001).
**Lim, 2014; Singapore**	Orthopedic, urologic, general surgery (n=170, 85 each)	Spinal (US vs. LM group)	No differences in first attempt success rate (US: 64% *vs.* LM: 52%), number of needle redirections, and complications (paresthesia, bloody tap). Fewer supervisor interventions (p=0.03) and higher patient satisfaction (p<0.001) in the US group. Needling time was also shorter (2.9±3.6 *vs.* 3.9±3.7 minutes; p=0.007)
**Sahin, 2014; Turkey**	Obstetrics non-obese and obese (BMI>30) (n=100, 4 groups, 25 each group)	Spinal at L4-5 (US vs. LM group subdivided into lean and obese (BMI >30kg/m2) – 4 groups)	Fewer attempts and fewer needle insertions were detected in US groups (p<0.001). First attempt success rate under US guidance was 92 % in comparison to 44 % using a LM technique in obese parturients (p<0.001). Needling time was shorter in US groups (22 vs. 52 s, p = 0.031). No differences in complication rates. *[obese subgroups 25 vs 25: failure rate 2/25 vs 2/25; first attempt 23/25 vs 11/25; needling time 37.2 ± 35.6 vs 150 ± 211.8 (s)=]*
**Srinivasan, 2015; Ireland**	Orthopedic (THR, TKR) (n=100, 50 each)	Spinal (US vs LM group)	The US group had fewer needle redirections (4.0±4.0 *vs.* 8.2±12.3; p = 0.01) and punctures (1.28±0.7 *vs.* 1.98±1.66; p=0.0021). All other parameters, including grading of palpated landmarks, time taken for spinal anesthetic injection, periprocedural pain scores, periprocedural patient discomfort visual analog scale score, conversion to general anesthetic, paresthesia, and radicular pain during needle insertion, were similar between the 2 groups.
**Srinivasan, 2018; Ireland**	Orthopedic (THR, TKR) (n=119, US 59, LM 60)	Spinal (US guided paramedian spinal L5-S1 vs LM group - best	There was no difference in number of redirections/insertions between the two groups. The first pass success rates (1 needle insertion and 1 needle direction) was significantly greater in Group LM compared to Group US (43% vs. 22%, p= 0.02).

		interspace selected)	
**Turkstra, 2017; Canada**	Obstetrics CS (n=80, 40 each)	Spinal (US vs LM group)	Number of attempts (insertions/redirections) was 3 (2-7) for the US group and 3 (1-60) for the LM group (p=0.69). Needling time was 92 (51-140) seconds vs 75 (53-126) seconds in the LM group (p=0.57). There was no statistical difference between the groups in need for staff intervention, paresthesia, bloody tap, lumbar interspace, or block height.
**Urfalioglu, 2017; Turkey**	Obstetrics CS, obese (n=97, US 48, LM 49) (BMI pre and post >30)	Spinal L4-5 (US vs. LM group)	The numbers of needle insertions and redirections were significantly fewer in the US than the LM group (p<0.001). Procedure time was significantly longer in the US than in the LM group (8±2 and 5±1; respectively p<0.001). Needling time was similar between the two groups (p=0.063). No other intergroup differences were registered.
**Epidural Anesthesia**			
**Arzola, 2015; Canada**	Obstetrics labor anlagesia (n=128, US 60, LM 68)	Epidural (US *vs.* LM group)	No difference in median [IQR] epidural insertion time between US and LM group [174 (120 to 241) versus 180 (130 to 322.5) s]. Number of levels attempted/ needle redirections were similar in both groups. Total procedure time was longer in the US group. Failure rate and patient satisfaction was similar among groups.
**Balaban, 2017; Turkey**	Obstetrics labor analgesia (n=40, 20 each)	Epidural at L4-5 (US vs. LM group)	Number of needle insertions was 1.35±0.58 in US group and 1.2±0.4 in LM group. Number of levels was 1.05±0.22 in US group and 1.10 ±0.3 in LM group. Duration of epidural procedure was 93 seconds in ultrasound group and 88 seconds in control group. No statistically significant differences were found between the two groups. Sudden low back pain during needle insertion was significantly lower in the US group (p=0.03).

**Grau, AAS 2001; Germany**	Obstetrics labor analgesia (difficult epidural) (n=72, 36 each) difficult EDA: 2/3 parturients had either a history of difficult epidural anaesthesia (36%) or substantial alterations of the lumbar spine such as scoliosis, kyphosis or hyperlordosis (26%). 1/3 or 38% percent had a BMI >33 kg/m2	Epidural (US vs. LM group)	Number of needle insertions for the LM and US group were 2.6±1.4 *vs.*1.5±0.9 (p<0.001). Nmber of levels attempted were 1.5±0.7 for the LM group and 1.3±0.5 for the US group (p<0.05). Catheter advancement attempts (1.1±0.6 *vs.* 1.3±0.6; P<0.003), and VAS scores (0.8±1.4 *vs.* 1.8±2.7; p<0.035) were lower in the US group. The LM group had higher patient satisfaction scores (2.1±1.3 *vs.* 1.3±0.5; P<0.006). Failure rate, incidence of headache or backache did not differ significantly.
**Grau, 2002; Germany**	Obstetrics (n=300) (each group 85 labor, 65 CS, 150 each)	Epidural (US vs. LM group)	In the US group number of redirections were lower (1.3±0.6 *vs.* 2.2±1.1; p<0.013), fewer intervertebral levels punctured before success (1.1±0.4 *vs.* 1.3±0.6; p<0.029), fewer attempts to thread the catheter (1.3±0.6 *vs.* 2.1±1.1; P<0.001), lower maximal VAS pain score during labor/CS (0.8±1.5 *vs.* 1.3±2.2; p<0.006), fewer instances of incomplete anesthesia (p<0.03), and lower incidence of side effects (headache, backache) (p<0.011)
**Kawaguchi, 2011; Japan**	Orthopedics (Total hip arthroplasty) (n=24, 12 in each group)	Epidural (US vs. LM group)	Successful ipsilateral-dominant block were 83% in the US group and 17% in the LM group (P<0.004). No failure rate was reported. Sensory and motor functions on the non-operated side in the US group were significantly better maintained than those on the operated side and compared with those on the non-operated side in the LM group (P< 0.05). Pain scores at mobilisation, incidence of adverse events and use of supplemental analgesics were significantly lower in the ultrasound group than the LM group (P< 0.05).
**Malik, 2018; USA**	Obstetrics, labor analgesia (n=96, 47 US, 49 LM)	Epidural at L5-S1 vs L2-L4 (US *vs.* LM group)	No differences in primary and secondary endpoints between the two groups. Two catheters were replaced in the LM group and 1 in the US group.

**Perna, 2017; Italy**	Obstetrics labor analgesia (n=60, US 30, LM 30)	Epidural to L3-4 or L2-3 (US vs. LM group)	In the US group the number of attempts was lower than the LM group (1.70±0.87 *vs.* 3.43±3.8, p=0.019). Number of insertions was >1 in 23.3% in the LM group and number of redirections was >2 in 20% cases in the LM group. In the US group none of the patients required >1 reposition and >2 redirections of the needle (p=0.031 for both).
**Vallejo, 2010; USA**	Obstetrics labor analgesia (n=370 US 189, LM 181)	Epidural L3-4 or L4-5 (US vs. LM group)	The US group had lower rate of failed epidural technique (1.6% *vs.* 5.5%; p<0.02) and fewer needle redirections/ insertions [1 (1-6) *vs.* 2 (1-6); p<0.01). No significant differences were noted with respect to staff interventions, or accidental dural punctures.
**Wilkes, 2017; USA**	Obstetrics (n=50, US 22, US-sham 28)	Epidural L2-3, L3-4, L4-5, L5-S1 (US vs. US-Sham group)	Epidural placement decreased PPT in US (68%) and US sham (79%) groups. Number of redirections were reduced in the US group (US: 0.81±1.4, US-Sham: 1.58±2.1; p=0.04). Number of reinsertions were less in the US group (0.09±.23) compared to the US-Sham (2.18±2.3) group (p<0.001).
**Combined spinal epidural anesthesia**			
**Chin, 2018; Australia**	Obstetrics, CS (n=215, US 105, LM 110)	CSE below L1-2 (US vs. LM group)	First-attempt success was achieved in 67 (63.8%) and 42 (38.2%) women in the US and LM groups, respectively (adjusted p = 0.001). CSE was 'difficult' in 19 (18.1%) and 33 (30.0%) women in the US and LM groups, respectively (p = 0.09). Secondary outcomes did not differ significantly.
**Grau, RAPM 2001; Germany**	Obstetrics CS, (n=80, 40 each)	CSE L3-4 (US vs. LM group)	First attempt success was higher in the US group than the LM group (75% *vs.* 20%, p<0.001). Number of levels attempted was lower in the US group (p<0.039). Preparation time was similar between groups.
**Grau, 2004; Germany**	Obstetrics (n=30, 10 in each group)	CSE (Real time US vs. pre-procedural US vs. LM group, 3 groups)	Real time and preprocedural US group: fewer needle passes compared to control group (p=0.036), fewer redirections. No intergroup differences in patient satisfaction, VAS scores, incomplete analgesia or complications.

**Nassar, 2014; Egypt**	Obstetrics, Labor analgesia (n=110, 55 each)	CSE (US vs. LM group)	The US group had higher first attempt success rate (62.3% *vs.* 40.0%; p=0.037), fewer needle insertions (1.2±0.6 *vs.* 2.3±0.8; p=0.037), and fewer needle redirections (2.8±1.6 *vs.* 1.4±0.5; p<0.001). No significant difference between groups in total procedure time.
**Tawfik, 2017; Egypt**	Obstetrics CS (n=108, 53 US, 55 LM)	CSE at L2-3 or L3-4 (US vs. LM group)	The rate of successful epidural catheterization at the first needle directions was 60% in the LM group and 58.5% in the US group. No significant differences between the 2 groups in first attempt success rate, number of needle redirections and insertions, or patient satisfaction. The median (range) duration of the epidural procedure was 185 (57–680) seconds in the US group and 215 (114–720) seconds in the LM group (p=0.036) The overall rate of complications of the procedure was low in both groups.
**Wang, 2012; China**	Obstetrics CS, obese BMI>30 (n=60, 30 each)	CSE at L3-4 (US vs. LM group)	Higher first attempt success rate (100% *vs.* 70%; P=0.004) and fewer needle insertions (P=0.035) in the US group. The LM group demonstrated shorter CSE procedure time: 9.37±1.35 *vs.* 7.67±1.52 *vs.* minutes; P<0.037. Puncture site hemorrhage was similar in both groups.
**Lumbar Puncture**			
**Lahham, 2016; USA**	ER (n=158) (US 71, LM 87)	LP (US *vs.* LM group)	No signifi cant difference was found in procedure time, number of of needle redirections, no. of needle reinsertions between the two groups. In the LM group, four LPs were unsuccessful and in the POCUS group, only seven LPs were unsuccessful. Number of needle passes US: 4 (1–7) vs LM: 4 (1–8). Procedure time US: 195 (110–436) vs LM: 181 (73–517) seconds.
**Mofidi, 2013; Iran**	ER (n=80, 40 each) (different BMI subgroups; BMI<25 6US, 6LM; BMI 25-29 20US, 22LM; BMI>29 14US, 12LM)	LP (US vs. LM group)	Procedure time (LM 6.4 ± 1.2 *vs.* US: 3.3 ± 1.2, p=0.032) and pain scores (LM 7.4 ± 1.1 *vs.* 4.4 ± 1.4, p=0.001) were lower in the US group. Number of attempts and number of traumatic LPs were significantly lower in US group (p=0.047 and p=0.024). In patients with different subgroups of BMI, US-guided LP showed better

			results and less complications when compared with the LM technique. *[obese BMI>29 US = 14, LM=12, 3.9 ±1.2 vs 9.1±1.1 min procedure time and number of attempts 1.72± 1.65 vs 3.36±2.51]*
**Nomura, 2007; USA**	ER (n=46, US 24, LM 22) (subgroup of 12 pts BMI > 30kg/m2, US=5, LM=7)	LP (US vs. LM group)	Failure rate was higher in the LM group (6/22 *vs.* 1/24) (RR, 1.32; 95%CI: 1.01–1.72). Number of attempts was not different between groups. *[obese (BMI>30) US=5, LM=7 and 4/7 LM attempts failed versus 0/5 US attempts (RR, 2.33; 95%CI: 0.99–5.49), procedure time 20.3US vs 25.3LM]* The ease of the procedure was better with ultrasound.
**Peterson, 2014; USA**	ER (n=100) [subgroup analysis of 51 pts with difficult landmarks (not palpable or difficult to palpate landmarks)]	LP (US vs. LM group)	No significant differences between the US group and the LM group for both primary and secondary outcomes *[difficult spine passes 4 ± 3.2 vs 7.4 ± 9.4, failure rate 5/22 vs 11/29, needling time 1.6 (1.2–3.2) 2.0 (1.1–5.0) =].*

Supplementary Figure 1: Risk of bias overview

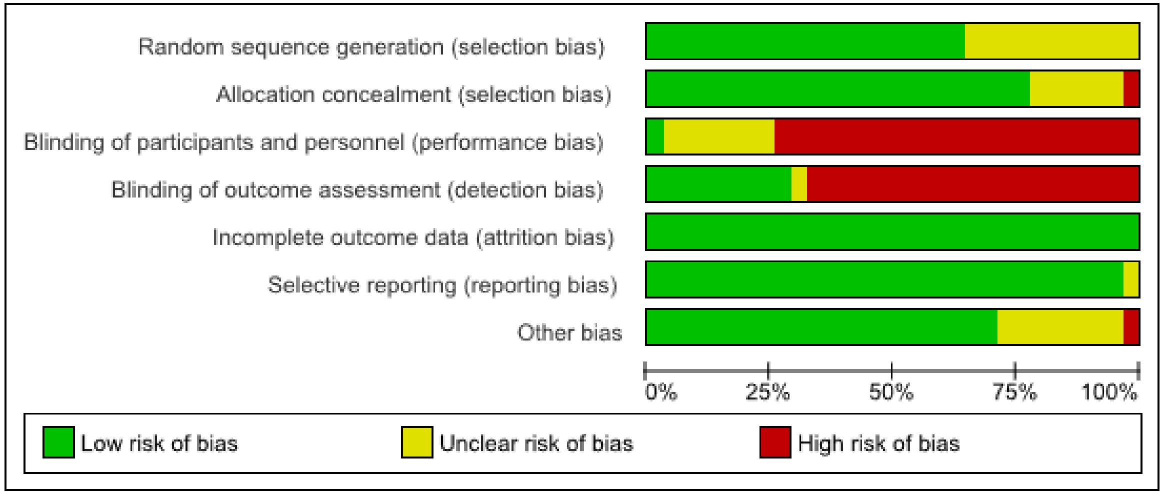

Supplementary Figure 2A, 2B: **Analysis of technical failure - Funnel Plots and Publication Bias**

In all patients we checked the publication bias concerning failure rate, the funnel plot showed some asymmetry (S Figure 2A), however with a minor effect on the effect estimate as indicated by the trim and fill analysis (estimated RR = -0.29 [99% CI, -0.69 to 0.11], number of missing studies = 1). However, neither Begg and Mazumdar rank correlation test (p = 0.298) nor Egger's regression asymmetry test (p = 0.552) confirmed the presence of significant publication bias. In difficult spine/obese trials, the funnel plot showed some asymmetry (S Figure 2B), however with a minor effect on the effect estimate as indicated by the trim and fill analysis (estimated RR = -0.36 [99% CI, -1.23 to 0.5], number of missing studies = 2). However, neither Begg and Mazumdar rank correlation test (p = 0.938) nor Egger's regression asymmetry test (p = 0.727) confirmed the presence of significant publication bias.

Publication bias analysis was performed using Meta-Essentials (Suurmond R, van Rhee, H, Hak T. Introduction, comparison and validation of Meta-Essentials: A free and simple tool for meta-analysis. Research Synthesis Methods. 2017:1-17. doi.org/10.1002/jrsm.1260).

S Figure 2A *(left)– Funnel plot of all trials (blue circles) and Trim and Fill analysis*

S Figure 2B *(right)– Funnel plot of trials (blue circles) and Trim and Fill analysis with 2 missing trials (orange circles)*

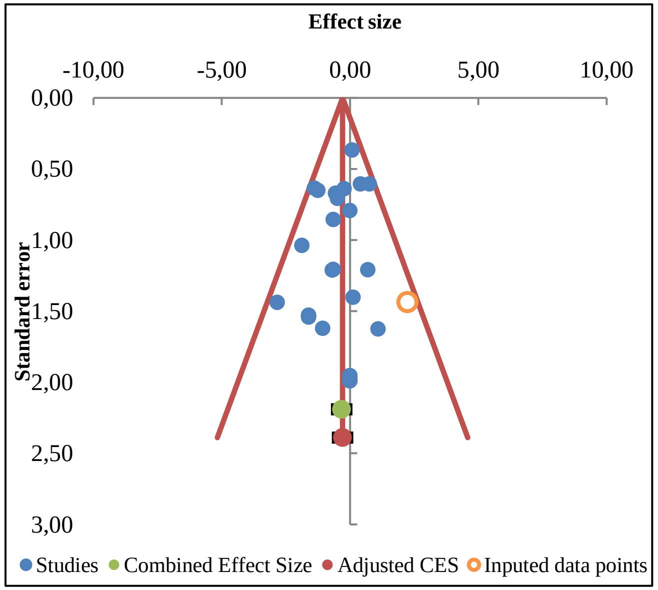

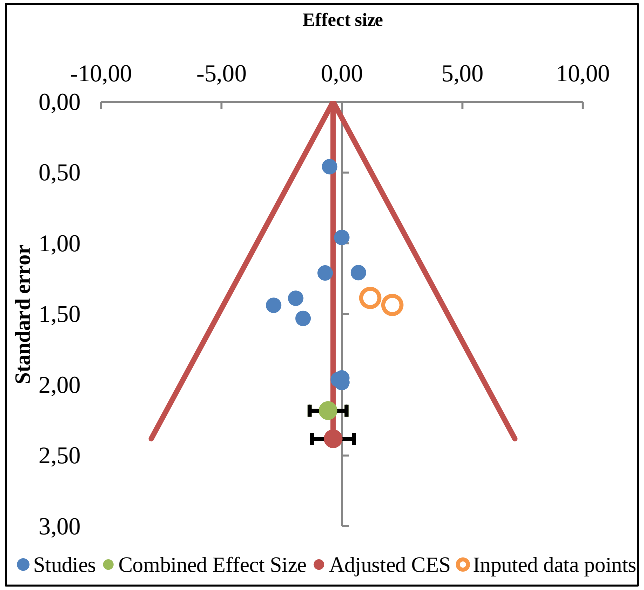

Supplementary Figures 3A, 3B: Analysis of first attempt success rate - Funnel Plots and Publication Bias

In all patients we checked the publication bias concerning first attempt success rate, the funnel plot showed some asymmetry (S Figure 3A), however with a minor effect on the effect estimate as indicated by the trim and fill analysis (estimated RR = 0.41 [99% CI, 0.14 to 0.68], number of missing studies = 0). However, neither Begg and Mazumdar rank correlation test (p = 0.221) nor Egger's regression asymmetry test (p = 0.374) confirmed the presence of significant publication bias. For difficult spine and obese patients subgroup the funnel plot was symmetrical (eFigure 5B) with trim and fill analysis (estimated RR = 0.61 [99% CI, 0.3 to 0.92], number of missing studies = 0). Begg and Mazumdar rank correlation test (p = 0.624) and Egger's regression asymmetry test (p = 0.682) confirmed the absence of significant publication bias.

S Figure 3A*(left) – Funnel plot of trials (blue circles) and Trim and Fill analysis*

S Figure 3B *(right)– Funnel plot of difficult spine and obese patients trials (blue circles) and Trim and Fill analysis*

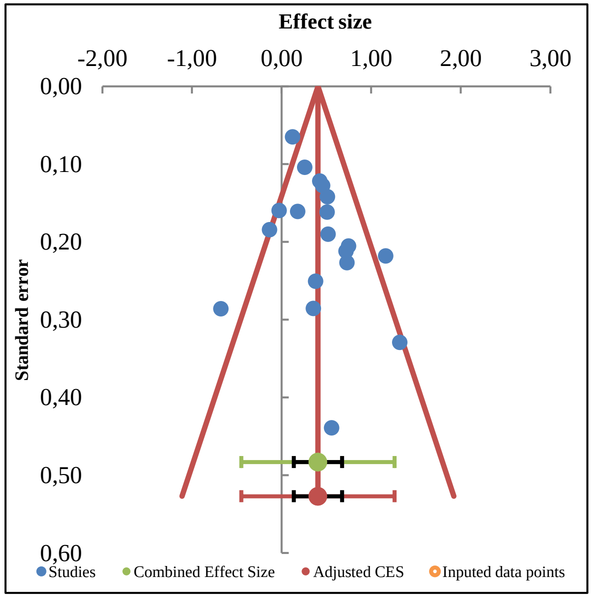

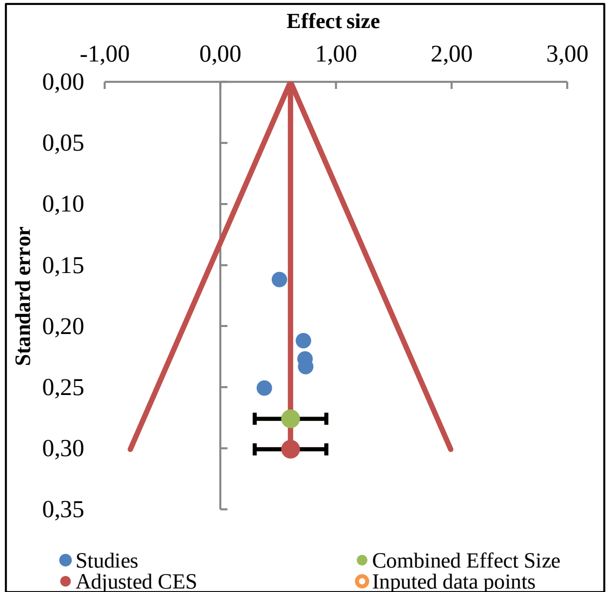

Supplementary Figures 4A, 4B: Analysis of number of needle redirections - Funnel Plots and Publication Bias

In all patients we checked the publication bias concerning first attempt success rate, the funnel plot showed some asymmetry (S Figure 4A), however with a minor effect on the effect estimate as indicated by the trim and fill analysis (estimated MD = 0.55 [99% CI, 0.28 to 0.83], number of missing studies = 0). However, neither Begg and Mazumdar rank correlation test (p = 0.494) nor Egger's regression asymmetry test (p = 0.515) confirmed the presence of significant publication bias.

For difficult spine and obese patients' subgroup the funnel plot showed some asymmetry (S Figure 4B), however with a minor effect on the effect estimate as indicated by the trim and fill analysis (estimated MD = 0.84 [99% CI, 0.54 to 1.15]). However, neither Begg and Mazumdar rank correlation test (p = 0.532) nor Egger's regression asymmetry test (p = 0.772) confirmed the presence of significant publication bias.

***S Figure 4A** (left)– Funnel plot of trials (blue circles) and Trim and Fill analysis*

***S Figure 4B** (right)– Funnel plot of difficult spine and obese patients trials (blue circles) and Trim and Fill analysis*

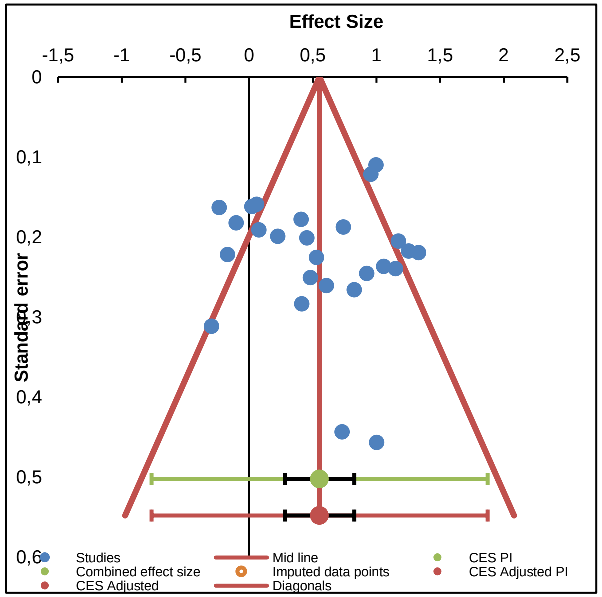

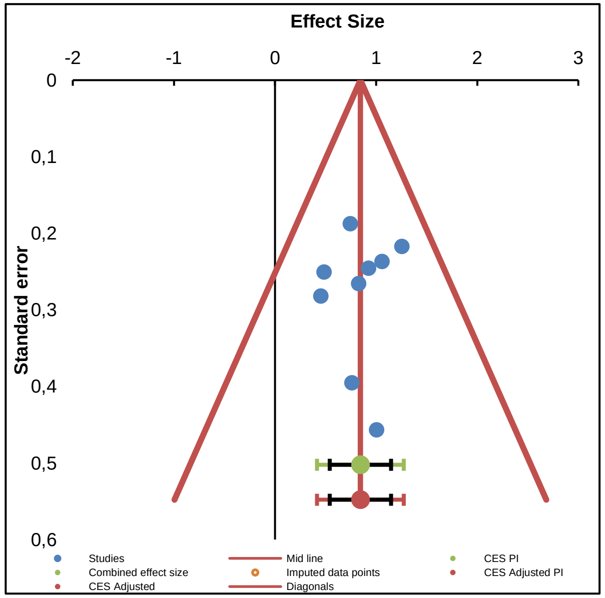

Supplementary Figures 5A, 5B: Analysis of trials reporting total procedure time

Total procedure time (time to identify landmarks and needling time) was reported in 11 RCTs. The standardized mean difference (SMD) was 0.80 (99% CI, -0.38 to 1.97), Z=1.75, p=0.08, p for heterogeneity < 0.0001, I^2 = 97%, Q = 375.25 (S Figure 5A). Seven RCTs reported data on difficult spine and obese patients (S Figure 5B). The SMD for these 7 trials was 0.23 [99% CI -1.10 to 1.56), Z=0.45, p=0.66, p for heterogeneity < 0.0001, I^2 = 96% and Q = 144.96.

*S Figure **5A** Analysis of trials concerning total procedure time and **5B** trials involving difficult spine and obese patients*

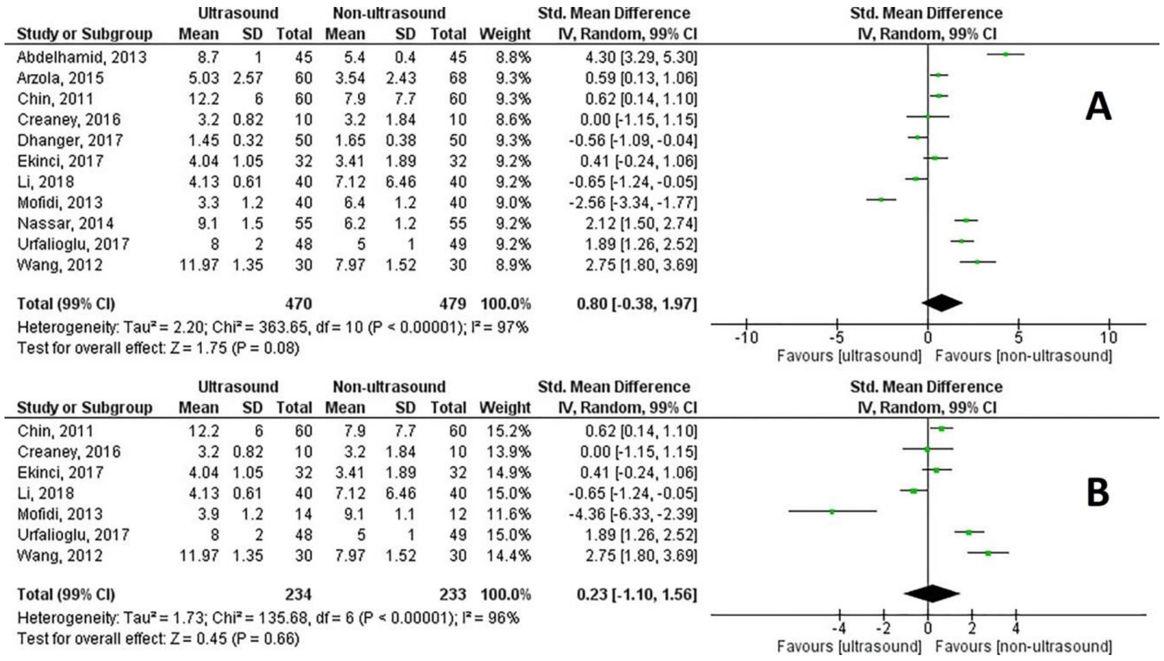

Supplementary Figures 6A, 6B: Analysis of trials reporting needling time

Needling time (time from when needle touched the skin until the desired outcome was obtained) was reported in 16 trials with a SMD of -0.33 (99% CI, -0.74 to 0.09), Z=2.02, p=0.04, p for heterogeneity < 0.0001, I^2 = 89% and Q = 144.30 (S Figure 6A) while data from 7 RCTs involving difficult spine and obese patients only had a SMD of -0.23 (99% CI, -0.85 to 0.39), Z=0.96, p=0.34, p for heterogeneity < 0.0001, I^2 = 84% and Q=37.95 (S Figure 6B).

*S Figure **6A** Analysis of trials concerning needling time and **6B** trials involving difficult spine and obese patients*

	Ultrasound			Non-Ultrasound				Std. Mean Difference
Study or Subgroup	Mean	SD	Total	Mean	SD	Total	Weight	IV, Random, 99% CI
Abdelhamid, 2013	3.9	0.23	45	4.6	0.5	45	6.1%	-1.78 [-2.43, -1.14]
Ansari, 2014	0.69	0.745	75	0.875	0.93	75	6.7%	-0.22 [-0.64, 0.20]
Arzola, 2015	2.98	1.53	60	3.54	2.43	68	6.6%	-0.27 [-0.73, 0.19]
Balaban, 2017	1.87	1.2	20	1.71	0.9	20	5.6%	0.15 [-0.67, 0.96]
Chin, 2011	5	4.9	60	7.3	7.6	60	6.6%	-0.36 [-0.83, 0.12]
Creaney, 2016	1.67	0.9	10	2.66	1.73	10	4.5%	-0.69 [-1.88, 0.51]
Dhanger, 2017	0.53	0.1	50	0.86	0.2	50	6.1%	-2.07 [-2.71, -1.43]
Lahham, 2016	4.17	4.1	71	4.35	5.57	87	6.7%	-0.04 [-0.45, 0.38]
Li, 2018	0.77	0.26	40	3.04	3.89	40	6.3%	-0.82 [-1.42, -0.21]
Lim, 2014	2.9	3.6	85	3.9	3.7	85	6.7%	-0.27 [-0.67, 0.12]
Nassar, 2014	6.1	0.7	55	5.8	1	55	6.5%	0.35 [-0.15, 0.84]
Peterson, 2014	1.8	1.52	38	2.54	3.3	39	6.3%	-0.28 [-0.87, 0.31]
Tawfik, 2017	3.42	1.8	53	4.05	1.91	55	6.5%	-0.34 [-0.84, 0.16]
Turkstra, 2017	1.575	1.14	40	1.42	0.935	40	6.3%	0.15 [-0.43, 0.72]
Urfalioglu, 2017	4	1	48	4	1	49	6.5%	0.00 [-0.52, 0.52]
Wang, 2012	9.37	1.35	30	7.67	1.52	30	5.9%	1.17 [0.44, 1.89]
**Total (99% CI)**			**780**			**808**	**100.0%**	**-0.33 [-0.74, 0.09]**

Heterogeneity: Tau² = 0.36; Chi² = 140.69, df = 15 (P < 0.00001); I² = 89%
Test for overall effect: Z = 2.02 (P = 0.04)

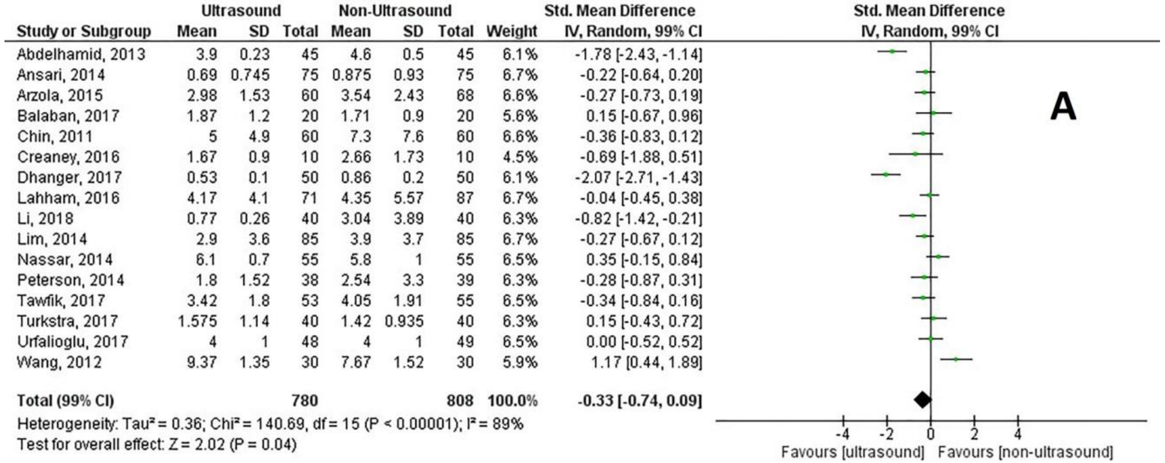

	Ultrasound			Non-Ultrasound				Std. Mean Difference
Study or Subgroup	Mean	SD	Total	Mean	SD	Total	Weight	IV, Random, 99% CI
Chin, 2011	5	4.9	60	7.3	7.6	60	16.0%	-0.36 [-0.83, 0.12]
Creaney, 2016	1.67	0.9	10	2.66	1.73	10	10.7%	-0.69 [-1.88, 0.51]
Li, 2018	0.77	0.26	40	3.04	3.89	40	15.2%	-0.82 [-1.42, -0.21]
Peterson, 2014	2.03	1.6	22	2.75	3.04	29	14.2%	-0.28 [-1.01, 0.45]
Sahin, 2014	0.62	0.6	25	2.5	3.53	25	14.0%	-0.73 [-1.49, 0.02]
Urfalioglu, 2017	4	1	48	4	1	49	15.7%	0.00 [-0.52, 0.52]
Wang, 2012	9.37	1.35	30	7.67	1.52	30	14.2%	1.17 [0.44, 1.89]
**Total (99% CI)**			**235**			**243**	**100.0%**	**-0.23 [-0.85, 0.39]**

Heterogeneity: Tau² = 0.33; Chi² = 36.77, df = 6 (P < 0.00001); I² = 84%
Test for overall effect: Z = 0.96 (P = 0.34)

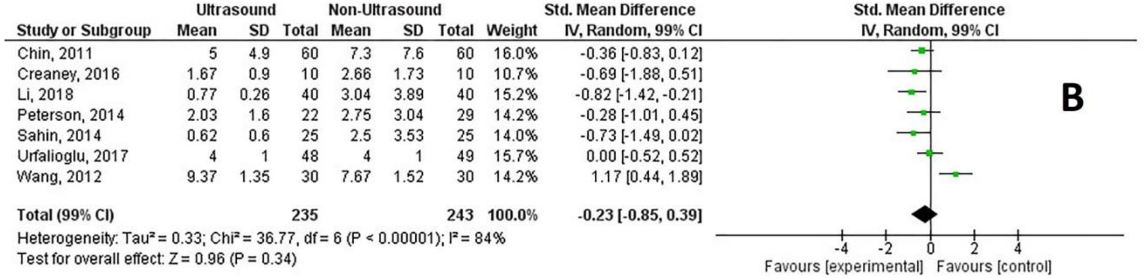

Supplementary Table 3: Quality of evidence for each outcome following the GRADE Working Group system**.**

**Primary Outcome**	**Quality of evidence for all patients**	**Quality of evidence for difficult spine and obese patients**
Risk of Technical failure	High	High
First attempt success	Low	Moderate
Number of needle redirection	Low	Low
Procedure Time	Very low	Very low
Needling Time	Very low	Very low

Supplementary Table 3: GRADE score analysis

**Primary Outcome**	**Risk of bias**	**Large effect**	**Dose response**	**Residual confounding**	**Inconsistency**	**Indirectness**	**Imprecision**	**Pub bias**	**Total**
Risk of Technical failure	-1	**+2**	-	-	0	-	-	-	+1
First attempt success	-1	+1	-	-	-2	-	-	-	-2
Number of needle redirection	-1	+2	-	-	-2	-	-1	-	-3
Procedure Time	-1	+1	-	-	-2	-	-1	-	-3
Risk of Technical failure OB	-1	**+1**	-	-	0	-	-	-	0
First attempt success OB	-1	-	-	-	0	-	-	-	-1

Number of needle redirection OB	-1	-	-	-	0	-	-1	-	-2
Procedure Time OB	-1	-	-	-	-2	-	-2	-	-5

Supplementary Table 4: EQUATOR Checklist

Section/topic	#	Checklist item	Reported on page #
**TITLE**			
Title	1	Identify the report as a systematic review, meta-analysis, or both.	1
**ABSTRACT**			
Structured summary	2	Provide a structured summary including, as applicable: background; objectives; data sources; study eligibility criteria, participants, and interventions; study appraisal and synthesis methods; results; limitations; conclusions and implications of key findings; systematic review registration number.	2
**INTRODUCTION**			
Rationale	3	Describe the rationale for the review in the context of what is already known.	3-4
Objectives	4	Provide an explicit statement of questions being addressed with reference to participants, interventions, comparisons, outcomes, and study design (PICOS).	3-4
**METHODS**			
Protocol and registration	5	Indicate if a review protocol exists, if and where it can be accessed (e.g., Web address), and, if available, provide registration information including registration number.	4
Eligibility criteria	6	Specify study characteristics (e.g., PICOS, length of follow-up) and report characteristics (e.g., years considered, language, publication status) used as criteria for eligibility, giving rationale.	4-5

Information sources	7	Describe all information sources (e.g., databases with dates of coverage, contact with study authors to identify additional studies) in the search and date last searched.	4-5
Search	8	Present full electronic search strategy for at least one database, including any limits used, such that it could be repeated.	Supp file pg 2
Study selection	9	State the process for selecting studies (i.e., screening, eligibility, included in systematic review, and, if applicable, included in the meta-analysis).	5
Data collection process	10	Describe method of data extraction from reports (e.g., piloted forms, independently, in duplicate) and any processes for obtaining and confirming data from investigators.	5-6
Data items	11	List and define all variables for which data were sought (e.g., PICOS, funding sources) and any assumptions and simplifications made.	5-6
Risk of bias in individual studies	12	Describe methods used for assessing risk of bias of individual studies (including specification of whether this was done at the study or outcome level), and how this information is to be used in any data synthesis.	6-7
Summary measures	13	State the principal summary measures (e.g., risk ratio, difference in means).	7
Synthesis of results	14	Describe the methods of handling data and combining results of studies, if done, including measures of consistency (e.g., I^2) for each meta-analysis.	7
**Section/topic**	**#**	**Checklist item**	**Reported on page #**
Risk of bias across studies	15	Specify any assessment of risk of bias that may affect the cumulative evidence (e.g., publication bias, selective reporting within studies).	6-7
Additional analyses	16	Describe methods of additional analyses (e.g., sensitivity or subgroup analyses, meta-regression), if done, indicating which were pre-specified.	7
**RESULTS**			
Study selection	17	Give numbers of studies screened, assessed for eligibility, and included in the review, with reasons for exclusions at each stage, ideally with a flow diagram.	8, fig1
Study characteristics	18	For each study, present characteristics for which data were extracted (e.g., study size, PICOS, follow-up period) and provide the citations.	8-9
Risk of bias within studies	19	Present data on risk of bias of each study and, if available, any outcome level assessment (see item 12).	9, table 1, fig 2, suppl

			file efig1,
Results of individual studies	20	For all outcomes considered (benefits or harms), present, for each study: (a) simple summary data for each intervention group (b) effect estimates and confidence intervals, ideally with a forest plot.	table 1, suppl file etable 1
Synthesis of results	21	Present results of each meta-analysis done, including confidence intervals and measures of consistency.	9-12, fig 3, fig4, fig 5, suppl file efig 5, efig6
Risk of bias across studies	22	Present results of any assessment of risk of bias across studies (see Item 15).	pg 9-12, suppl file efig 2, efig3, efig 4
Additional analysis	23	Give results of additional analyses, if done (e.g., sensitivity or subgroup analyses, meta-regression [see Item 16]).	9-12, fig 3, fig4, fig 5, suppl file efig 5, efig6
**DISCUSSION**			
Summary of evidence	24	Summarize the main findings including the strength of evidence for each main outcome; consider their relevance to key groups (e.g., healthcare providers, users, and policy makers).	13,14
Limitations	25	Discuss limitations at study and outcome level (e.g., risk of bias), and at review-level (e.g., incomplete retrieval of identified research, reporting bias).	15, 16
Conclusions	26	Provide a general interpretation of the results in the context of other evidence, and implications for future research.	14-16
**FUNDING**			
Funding	27	Describe sources of funding for the systematic review and other support (e.g., supply of data); role of funders for the systematic review.	1
